# Heterosubtypic cross‐protection induced by whole inactivated influenza virus vaccine in mice: influence of the route of vaccine administration

**DOI:** 10.1111/irv.12142

**Published:** 2013-09-16

**Authors:** Natalija Budimir, Aalzen de Haan, Tjarko Meijerhof, Emma Gostick, David A. Price, Anke Huckriede, Jan Wilschut

**Affiliations:** ^1^ Department of Medical MicrobiologyMolecular Virology SectionUniversity of GroningenUniversity Medical Center GroningenGroningenThe Netherlands; ^2^ Institute of Infection and ImmunityCardiff University School of MedicineCardiffUK

**Keywords:** Cross‐protection, cytotoxic T lymphocytes, influenza, mucosal vaccination, parenteral vaccination, whole inactivated virus

## Abstract

**Background:**

Development of influenza vaccines capable of inducing broad protection against different virus subtypes is necessary given the ever‐changing viral genetic landscape. Previously, we showed that vaccination with whole inactivated virus (WIV) induces heterosubtypic protection against lethal virus infection in mice. Whole inactivated virus‐induced cross‐protection was found to be mediated primarily by flu‐specific CD8+ T cells.

**Objectives:**

As it has been demonstrated that the route of vaccine administration strongly influences both the quantity and quality of vaccine‐induced immunity, in this study, we determined which route of WIV administration induces optimal heterosubtypic cross‐protection.

**Methods:**

We compared the magnitude of the immune response and heterosubtypic protection against lethal A/PR/8/34 (H1N1) infection after subcutaneous (SC), intramuscular (IM), and intranasal (IN) vaccination with A/NIBRG‐14 (H5N1) WIV.

**Results:**

Subcutaneous and IM administration was superior to IN administration of influenza WIV in terms of flu‐specific CD8+ T‐cell induction and protection of mice against lethal heterosubtypic challenge. Surprisingly, despite the very low flu‐specific CD8+ T‐cell responses detected in IN‐vaccinated mice, these animals were partially protected, most likely due to cross‐reactive IgA antibodies.

**Conclusion:**

The results of this study show that the magnitude of WIV‐induced flu‐specific CD8+ T‐cell activity depends on the applied vaccination route. We conclude that parenteral administration of WIV vaccine, in particular IM injection, is superior to IN vaccine delivery for the induction of heterosubtypic cross‐protection and generally appears to elicit stronger immune responses than mucosal vaccination with WIV.

## Introduction

Annual influenza epidemics represent a major cause of morbidity and mortality worldwide.^1^ Occasionally, as a result of antigenic shift, a new influenza virus subtype, which is not recognized by antibodies induced by previous infection or vaccination, appears in the human population. In the absence of specific immunity, such viruses can be transmitted rapidly to cause global pandemics.^2^

Influenza vaccines have substantially reduced the burden of disease due to influenza infection, especially in vulnerable groups, such as the elderly and patients with chronic respiratory or cardiovascular disease.^3^, ^4^ Although vaccination still represents the best way to prevent influenza, there is an urgent need for improvement. Current vaccination approaches aim to induce antibody responses against the variable viral surface antigens, mainly hemagglutinin (HA). Consequently, the overall success of seasonal vaccination depends mainly on the antigenic match between the vaccine and the circulating virus strain and may vary substantially from one season to the next.^5^, ^6^ The antigenic composition of an emerging pandemic virus cannot be predicted at all, which makes it difficult to prepare sufficient vaccine stocks in due time.^6^ To restrict the impact of “between‐season” strain variability and to attenuate the threat of a pandemic influenza outbreak, cross‐protective influenza vaccines are desirable. Such vaccines should ideally target conserved viral antigens, such as the internal nucleoprotein (NP) or the matrix protein (M1).^7^, ^8^

Previously, we demonstrated that vaccination with whole inactivated virus (WIV), but not with subunit or split‐virion vaccines, can protect mice from lethal heterosubtypic influenza challenge. This protection was due to the induction of a potent CD8+ T lymphocyte response against conserved virus proteins, such as NP.^9^

In addition to the nature of the antigen and the presence of an adjuvant, the route of administration can strongly influence the immunogenicity of a vaccine.^10^, ^11^, ^12^ For example, intranasally administered virus‐like particles (VLPs) expressing influenza M2 protein induce superior antibody responses compared with the same vaccine administered subcutaneously.^12^

Here, we investigated which route of WIV administration optimally induces heterosubtypic cross‐protection against influenza. Specifically, we compared parenteral routes of administration (subcutaneous, SC; intramuscular, IM) with a mucosal vaccination route (intranasal, IN). After administration of H5N1 WIV, we determined the survival of mice after heterosubtypic challenge with H1N1 virus and measured the magnitude of induced flu‐specific CD8+ T‐cell responses. The main finding of the study is that full protection against lethal heterosubtypic challenge in mice was obtained only when WIV was delivered through one of the parenteral routes. The protection correlated with the presence of flu‐specific CD8+ T cells. Only partial protection was observed in IN‐vaccinated mice, which mounted very poor flu‐specific CD8+ T‐cell responses but developed cross‐neutralizing IgA antibodies.

## Materials and methods

### Virus strains and vaccine preparation

Vaccine virus (NIBRG‐14/H5N1, a 6:2 reassortant strain of A/PR/8/34 and A/Vietnam/1194/2004) was cultured on Madin–Darby canine kidney (MDCK) cells. Challenge virus (A/PR/8/34 H1N1), cultured in eggs, was a kind gift from Solvay Biologicals (Weesp, the Netherlands). Whole inactivated virus vaccine was prepared by inactivation of the virus with 0·1% β‐propiolactone (BPL) for 24 hours at room temperature, followed by dialysis for 24 hours against HNE buffer (5 mm HEPES, 150 mm NaCl, 0·1 mm EDTA, pH 7·4). Inactivation of the vaccine was tested as described previously.^13^

### Vaccination and challenge

Mouse experiments were performed in accordance with Dutch legislation on animal experiments and were approved by the Ethics Committee on Animal Research of the University Medical Center Groningen (permit number: 5101B). Vaccination, blood sampling, challenge, and euthanasia were performed under isoflurane anesthesia.

Female C57BL/6 mice, 6–8 weeks old, were purchased from Harlan, the Netherlands. Each vaccination group consisted of 12 mice that were divided into two subgroups. One of the subgroups was monitored for body weight change over a period of 2 weeks post‐challenge. Mice from the other subgroup were euthanized 6 days post‐challenge for analysis of lung virus titers and immune response parameters. On days 0 and 21, mice received two doses of 20 μg of total WIV protein administered (i) SC, 200 μl in the neck area; or (ii) IM, 25 μl in the right hind leg, or (iii) IN, 40 μl equally distributed into both nostrils. Using this procedure, some part of the IN‐inoculated volume may distribute further down to the lower respiratory tract or the gastrointestinal tract. Non‐vaccinated mice served as controls. One week after the booster, mice were anesthetized and inoculated IN with 100 TCID_50_ of PR8 virus in a total volume of 40 μl. Viral TCID_50_ was determined according to a previously published protocol.^9^, ^14^ In brief, lungs were dissected, homogenized, and stored at −80°C until further use. Virus titers were determined by adding serial dilutions of the clarified homogenates to MDCK cells in 96‐well plates and culturing the cells in the presence of TPCK trypsin. The highest dilution of culture supernatant that still showed hemagglutination activity was taken as the virus titer in the lungs. Titers are indicated as 10 log virus titer per gram of lung tissue.

Upon challenge, mice were monitored for disease symptoms (i.e., ruffled fur and weight loss). Loss of more than 20% of the total body weight during the 2‐week post‐challenge period was an indication for euthanasia.

### Tetramer staining of blood, spleen, lymph node, and lung lymphocytes

Tetramer stainings on blood, spleen, lymph node, and lung lymphocytes were performed as described before.^9^ In brief, lymphocytes were isolated from the tissues using previously described methods^9^ and washed with FACS buffer (1% BSA, 5 mm EDTA in PBS). Cells were then stained with anti‐mouse CD8a‐APC antibody (ImmunoSource, Schilde, Belgium) and influenza NP_366‐374_‐tetramer‐PE.^9^ Dead cells were excluded using 7AAD viability solution (ImmunoSource). Flow cytometric analysis was performed using a FACS Calibur flow cytometer (BD Biosciences, Breda, the Netherlands).

### Influenza‐specific IgG and IgA ELISA

ELISA was performed as described before.^9^, ^13^ In brief, microtiter plates were coated overnight with 0·5 μg of influenza H5N1 (NIBRG‐14) or H1N1 (PR/8) WIV per well. Plates were then blocked, washed, and incubated with twofold serial dilutions of serum or vaginal wash samples for 1·5 hour at 37°C. After washing, plates were incubated for 1 hour with HRP‐conjugated goat anti‐mouse IgG or IgA antibody (Southern Biotech, Birmingham, AL, USA) followed by 30‐min staining with o‐phenylene‐diamine staining solution. The reaction was stopped by adding 50 μl per well of 2 m H2SO4, and the absorbance was read at 492 nm.

### Microneutralization assay

Microneutralization assays were performed as described before.^9^ In brief, twofold serial dilutions of sera were added to 50 TCID_50_ of A/PR/8 virus and incubated for 2 hours at 37°C. Mixtures of serum and virus were then added to MDCK cells in 96‐well plates. After 1‐hour incubation at 37°C, culture supernatants were replaced by medium supplemented with 6 μg/ml of TPCK trypsin and cells were incubated for an additional 72 hours. Supernatants were harvested and tested for hemagglutinating activity. The highest dilution of serum preventing virus infection was taken as the neutralizing titer.

### Statistical methods

To determine the differences between vaccination groups with respect to flu‐specific CD8+ T‐cell frequencies, influenza antibody titers, and lung virus titers, the Mann–Whitney *U*‐test with a confidence interval of 95% was used. A value of *P* < 0·05 was considered statistically significant and is designated in the figures with an asterisk. Double and triple asterisks indicate *P* values of <0·01 and <0·001, respectively.

## Results

### Heterosubtypic cross‐protection induced by WIV administered through different vaccination routes

**Figure 1:**
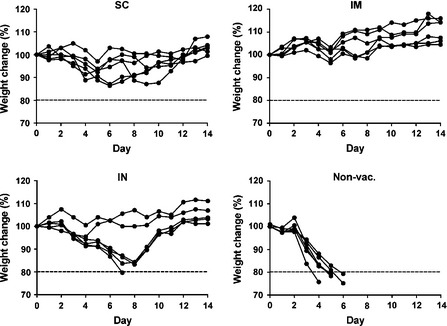
Body weight change and survival of WIV‐vaccinated mice after heterosubtypic challenge. Mice received two doses of 20 μg (total viral protein) NIBRG‐14 (H5N1) WIV, administered subcutaneously (SC), intramuscularly (IM), or intranasally (IN). Non‐vaccinated mice were used as controls. After the booster vaccination, mice were exposed to lethal heterosubtypic challenge with 100 TCID50 A/PR8 (H1N1) virus and monitored daily for body weight change over the following 2 weeks. Experiments were performed twice. A body weight loss of more than 20% was an indication for euthanasia (dashed line).

To evaluate the impact of different vaccination routes (SC, IM, and IN) on the efficacy of WIV‐induced heterosubtypic cross‐protection, mice were vaccinated twice with WIV derived from A/NIBRG‐14 (H5N1) virus and subsequently challenged with A/Puerto Rico/8/34 (PR8) influenza virus, an H1N1 strain. Seven days after the booster vaccination, mice were exposed to heterosubtypic challenge with 100 TCID50 of live PR8 and monitored daily for body weight loss. During this post‐challenge period, IM‐vaccinated mice experienced only minor body weight loss (Figure 1). Three of six SC‐vaccinated mice lost up to 12% of their body weight, after which they recovered. Four of six mice vaccinated via the IN route lost more than 15% of their body weight, and one of them had to be euthanized. Non‐immune mice experienced rapid weight loss and disease symptoms necessitating euthanasia by day 6 post‐challenge (Figure 1).

### Influence of the route of vaccine administration on lung virus clearance

**Figure 2:**
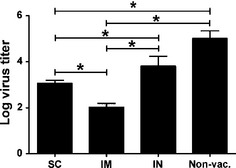
Lung virus titers after vaccination and heterosubtypic challenge. Virus titers in the lungs of mice vaccinated with H5N1 WIV, delivered via the indicated routes, and challenged with H1N1 virus, as in Figure 1, were measured 6 days post‐challenge. Experiments were performed twice. Bars represent mean titer±SEM of three mice per group. *P < 0·05; Mann–Whitney *U*‐test.

To investigate the influence of different administration routes on vaccine‐induced clearance of virus from the lungs, mice were vaccinated and challenged according to the schedule described above and sacrificed 6 days post‐challenge for lung viral titer assessment. Virus titers in the lungs of SC‐vaccinated mice were significantly lower compared with titers in lungs of non‐vaccinated mice. Vaccination with WIV via the IM route resulted in an even more pronounced decrease in titers (Figure 2). In contrast, virus titers in the lungs of IN‐vaccinated mice were not significantly reduced compared with titers measured in the lungs of non‐immune mice, although a trend toward lower titers was apparent (Figure 2). Fourteen days post‐challenge, virus titers in the lungs of all surviving mice were undetectable, irrespective of the route of vaccination (data not shown).

### Influence of the route of vaccine administration on WIV‐induced flu‐specific CD8+ T‐cell responses

Earlier, we showed that the main mediators of heterosubtypic cross‐protection induced in mice by SC administration of WIV are flu‐specific CD8+ T cells.^9^ Therefore, we evaluated the impact of different vaccine administration routes on the magnitude of the induced flu‐specific CD8+ T‐cell response. To this end, mice were vaccinated according to the schedule described above. One week after the booster vaccination, immediately prior to challenge, peripheral blood NP366‐374 tetramer+ CD8+ cells were measured. Six days post‐challenge, tetramer staining was also performed on cells from spleen, lungs, and lymph nodes draining the vaccine injection site (cervical for IN and SC injections; inguinal for IM injection).

**Figure 3:**
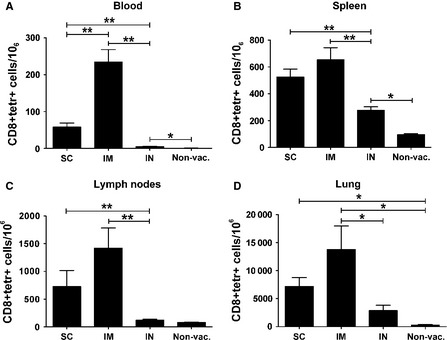
Flu‐specific CD8+ T‐cell responses induced by vaccination with WIV administered through different routes. Mice were vaccinated with H5N1 WIV via the indicated routes, as in Figure 1. Flu‐specific CD8+ T cells were measured by NP366‐374 tetramer staining in PBMCs pre‐challenge (A) and in spleens (B), local lymph nodes (C), and lungs (D) 6 days post‐challenge with H1N1 virus, as in Figure 1. Experiments were performed twice. Bars represent mean CD8+ tetramer+ cell numbers per 10^6^ cells ± SEM of six mice per group for PBMCs, spleens, and lymph nodes and three mice per group for lungs. *P < 0·05; *^*^ *P* < 0·01; Mann–Whitney *U*‐test.

Whole inactivated virus administered through the IM route induced significantly higher NP‐specific CD8+ T‐cell responses in blood, spleen, lung, and draining lymph nodes compared with levels induced by IN administration (Figure 3A–D). There were no significant differences in NP‐specific CD8+ T‐cell frequencies measured in the spleens, lungs, and lymph nodes of IM‐vaccinated and SC‐vaccinated mice on a per site basis (Figure 3B–D). However, in pre‐challenge blood, NP‐specific CD8+ T‐cell frequencies were significantly higher in IM‐vaccinated compared with SC‐vaccinated mice (Figure 3A). Vaccination via the IN route induced low numbers of NP‐specific CD8+ T‐cells, which only in spleen were significantly higher as compared to non‐vaccinated mice (Figure 3B). Thus, flu‐specific CD8+ T‐cell responses are induced more efficiently by parenteral compared with mucosal delivery of WIV.

### Influence of the route of vaccine administration on WIV‐induced antibody responses

To evaluate humoral immune responses induced by vaccination with WIV administered through different routes, we measured influenza‐specific IgG and IgA antibody titers in pre‐challenge blood. Additionally, antibody titers were measured in pre‐challenge vaginal washes; sampling of vaginal lavages provides access to a mucosal site without requiring euthanasia. It has been demonstrated that antibody titers (IgA) measured in vaginal washes correlate closely with antibody titers measured at other mucosal surfaces, including lung.^15^ Using ELISA, we assessed IgG and IgA levels specific for the vaccine virus (NIBRG‐14/H5N1) and for the challenge virus (PR8/H1N1). As a positive control for PR8‐specific antibodies, pooled sera of mice primed intraperitoneally with replicating PR8 virus were used.

**Figure 4:**
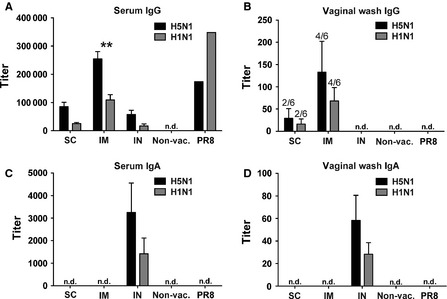
Total influenza‐specific systemic and mucosal IgG and IgA antibodies. ELISA plates were coated with vaccine (H5N1) or challenge (H1N1) virus. Titers of influenza‐specific IgG and IgA antibodies against both viruses were measured in pre‐challenge sera and vaginal washes obtained from mice vaccinated as in Figure 1. As a positive control, pooled sera obtained from mice primed intraperitoneally with live PR8 (H1N1) virus were used. Experiments were performed twice. Bars represent mean antibody titers ±SEM of six mice per group. *^*^ *P* < 0·01; Mann–Whitney *U*‐test.

The highest IgG antibody titers against homologous (H5N1) and heterologous (H1N1) virus were induced by IM administration of WIV (Figure 4A). In contrast, WIV administered through the SC or IN routes induced low‐serum IgG antibody titers against H5N1 virus and barely detectable titers against the H1N1 challenge virus (Figure 4A). A similar pattern was observed in vaginal washes. Four of six mice from the IM group had detectable mucosal IgG antibody titers against both the vaccine and the challenge virus; in contrast, only two of six mice from the SC group had detectable IgG titers and these were substantially lower (Figure 4B). Influenza‐specific mucosal IgG titers were not detectable in mice vaccinated through the IN route (Figure 4B).

In contrast to IgG antibodies, which were found in all vaccination groups (with the exception of vaginal washes from the IN group), IgA antibodies were detected only in sera and vaginal washes from IN‐vaccinated mice (Figure 4C, D).

### Virus neutralization by antibodies induced upon administration of WIV through different vaccination routes

**Figure 5:**
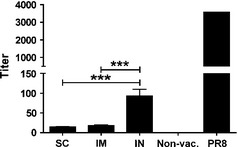
H1N1 virus‐neutralizing antibodies. Pre‐challenge sera from mice vaccinated with H5N1 WIV, administered through the indicated routes, as in Figure 1, were tested for the presence of antibodies capable of neutralizing the H1N1 challenge virus. As a positive control, pooled sera obtained from mice primed intraperitoneally with live PR8 (H1N1) virus were used. Experiments were performed twice. Bars represent mean titers ±SEM of six mice per group. ***P < 0·001; Mann–Whitney *U*‐test.

To determine whether antibodies induced by vaccination with WIV could potentially play a role in virus neutralization and protection from heterosubtypic challenge, we performed a microneutralization assay with PR8 virus using pre‐challenge sera and vaginal washes obtained from immunized animals. Sera from mice immunized through the SC or IM routes displayed only minimal neutralization activity against the PR8 challenge virus (Figure 5). In contrast, sera obtained from IN‐vaccinated mice neutralized the challenge virus with 10‐fold greater efficacy. None of the tested vaginal washes exhibited neutralizing activity, most likely due to antibody dilution (data not shown). Collectively, these observations suggest that IgA, but not IgG, antibodies could play a role in the partial heterosubtypic cross‐protection observed after IN vaccination.

## Discussion

In a previous study, we demonstrated that immunization of mice with influenza WIV vaccine provides protection against heterosubtypic challenge, primarily through the induction of cross‐reactive flu‐specific CD8+ T‐cell responses.^9^ Here, we show that parenteral administration, particularly IM injection, is superior to mucosal (IN) vaccine delivery for the induction of cross‐reactive flu‐specific CD8+ T‐cell responses and cross‐protection by WIV. However, mucosal immunization, as compared to IM or SC injection, induces a superior secretory IgA (SIgA) response, which may also contribute to heterosubtypic protection.

Our conclusion that parenteral vaccine administration is superior to mucosal delivery in terms of CD8+ T‐cell priming is consistent with observations in several other systems. For example, Bessa *et al*.^12^ showed that virus‐like particles containing a peptide derived from lymphocytic choriomeningitis virus induced a superior cellular immune response when the vaccine was administered through SC injection compared with mucosal administration. Furthermore, Decrausaz *et al*.^16^ observed that CD8+ T‐cell responses were induced more effectively by parenteral rather than mucosal administration of a human papillomavirus vaccine. In addition, in early studies on the induction of flu‐specific CD8+ T‐cell activity by isolated NP, the antigen was administered IM or SC.^17^

There are several potential explanations for the limited capacity of inactivated vaccines to induce CD8+ T‐cell responses when administered mucosally. First, antigens delivered to mucosal surfaces are likely to be diluted in mucosal secretions and or quickly removed, limiting their availability for immune recognition.^18^, ^19^ Importantly, low antigen availability is a limiting factor for cross‐presentation and CD8+ T‐cell induction.^20^ Also, antigen delivered through mucosal versus parenteral administration engages DCs with different cross‐presenting capacities.^21^, ^22^ Finally, mucosal immunization, as a consequence of the phenomenon of mucosal tolerance, may suppress the induction of systemic cellular responses.^23^, ^24^, ^25^

There appears to be a major difference between replicative and inactivated influenza vaccines in terms of their ability to induce CD8+ T‐cell responses upon mucosal delivery to the respiratory tract. Several studies in mice,^26^ ferrets,^27^ and non‐human primates^28^ have shown that mucosal administration, in particular pulmonary delivery,^29^ of infectious virus, resulting in a non‐lethal infection of the (lower) respiratory tract, is very effective at inducing robust CD8+ T‐cell responses and mediating cross‐protection upon subsequent challenge with divergent virus variants or subtypes.^30^ Indeed, establishment of a pulmonary infection appears to be more effective at inducing flu‐specific CD8+ T‐cell responses than priming via the intraperitoneal, intravenous, or IN routes.^29^ In contrast, mucosal delivery of inactivated vaccines induces mainly SIgA antibodies, but not flu‐specific CD8+ T‐cell responses.^31^ Presumably, the barriers that impede the efficient development of T‐cell responses upon mucosal delivery of inactivated vaccines do not affect replicative vaccines to the same extent. Productive infection results in the generation of comparatively high doses of viral antigen. In addition, active virus replication triggers DC activation, promotes the presentation of viral antigens in the context of both class I and class II MHC molecules, and may readily overcome mucosal tolerance.^32^ It is interesting in this respect that mucosal delivery of infectious influenza virus, as well as a recombinant adenovirus vector expressing influenza NP antigen, was more effective at inducing heterosubtypic cross‐protection than IM injection.^33^

Our finding that mucosal administration of influenza WIV vaccine is suboptimal for the induction of CD8+ T‐cell immunity is at variance with findings from Alsharifi *et al*.^34^ They compared heterosubtypic protection induced by γ ray‐inactivated WIV (γ‐WIV) using different administration routes and found that IN administration was superior to SC delivery. In our hands, however, SC injection of WIV induced solid cross‐protection that correlated closely with the magnitude of the NP‐specific CD8+ T‐cell response, while mucosal administration provided only partial protection that appeared to be mediated primarily by cross‐reactive SIgA. It is difficult to explain this apparent discrepancy. It is unlikely that the dose of antigen was lower in our study, although a direct comparison of doses cannot be made. Alsharifi *et al*. measured antigen dose in pfu equivalents, whereas we used protein concentration. Nonetheless, a conservative estimate would suggest that the antigen dose was substantially higher in our study. It is possible that the protection observed in the study by Alsharifi *et al*.^34^ was mediated by cross‐reactive antibodies, which were not investigated. Another important variable could be the use of different inactivation protocols for producing WIV; different inactivation protocols may yield vaccine formulations with varying capacities to activate cytosolic innate receptors and to induce cross‐protective T‐cell responses.^9^, ^35^ Also, it is possible that the γ‐WIV used by Alsharifi *et al*. was not entirely devoid of replication‐competent virus, which, as discussed above, is very efficient in inducing CD8+ T‐cell responses upon delivery to the respiratory tract. In this respect, it is interesting to note that infectivity of apparently completely inactivated γ‐WIV may be reconstituted through genetic complementation.^36^ Indeed, upon multiple infection of a single cell, viral particles critically damaged at different parts of the genome may complement each other thereby reconstituting the capacity to produce infectious virus particles. A similar phenomenon has recently been described in a study by Brooke *et al*.,^37^ showing that influenza virus often exists as a population of “abortive infectious forms” of virus that, through multiple infection, may reconstitute infectivity. A comparative study, involving head‐to‐head testing of similar doses of BPL‐inactivated WIV or γ‐WIV administered through different routes, would help to clarify the discrepancy between our findings and the findings by Alsharifi *et al*.^34^

Our data are also at apparent variance with those of Bodewes *et al*.^38^ In the present study, IM injection of WIV induced robust cross‐protective flu‐specific CD8+ T‐cell responses, whereas Bodewes *et al*. observed minimal flu‐specific CD8+ T‐cell induction and, as a result, no heterosubtypic cross‐protection after IM vaccination of mice with formaldehyde‐inactivated WIV.^38^ This apparent discrepancy may be explained by the use of divergent virus inactivation protocols and the use of different combinations of vaccine strain and challenge strain. As we showed previously,^9^, ^13^ virus inactivation procedures using formaldehyde severely compromise the membrane fusion activity of WIV particles, which results in a significant decrease in the CD8+ T‐cell‐priming capacity of the vaccine. We prepared WIV using BPL as the inactivating agent, which preserves viral membrane fusion activity and, consequently, the CD8+ T‐cell‐priming ability of the vaccine to a considerable extent.^9^ Another variable that could explain the variance of our findings with findings of Bodewes *et al*. is the use of different combinations of vaccine strain and heterosubtypic challenge strain. Bodewes *et al*. used a reassortant H3N2 vaccine strain and a H5N1 A/IND challenge strain,^38^ while we used a reassortant H5N1 vaccine strain and a H1N1 A/PR8 challenge strain. The H5N1 reassortant contains internal virus proteins derived from A/PR8. While this optimizes internal viral antigen recognition by flu‐specific CD8+ T cells, this model may not optimally reflect challenges that are faced in induction of cross‐protection in humans.

Although mucosal administration of inactivated vaccine was suboptimal for the induction of flu‐specific CD8+ T‐cell activity in our experiments, we did observe partial protection from heterosubtypic challenge in mice immunized IN with WIV. The observed protection correlated with the presence of SIgA antibodies in mucosal secretions and cross‐neutralizing serum antibodies which were found only after IN immunization, although it should be noted that the levels of antibodies in the vaginal washes might not fully reflect those of the respiratory organs. Sera of IM‐immunized mice contained only IgG antibodies and did not show any *in vitro* neutralizing capacity (Figures 4A, B and 5). These results are in agreement with observations of others. Indeed, several studies have demonstrated the induction of full or partial protection against homosubtypic or heterosubtypic influenza infection by vaccination through a mucosal route and have also shown a close correlation between protection and the presence of mucosal SIgA antibodies.^31^, ^39^, ^40^, ^41^ To establish whether a higher level of local protection (i.e., nasal cavity) is induced by IN immunization using WIV, a lower volume of challenge virus than used in the present study would be preferred. In this respect, aerosol inoculation of virus, for example, could mimicked natural influenza infection more closely.

In conclusion, the route of administration substantially influences the induction of cross‐reactive flu‐specific CD8+ T‐cell responses and heterosubtypic cross‐protection induced by influenza WIV in mice. Parenteral delivery of WIV, in particular IM vaccination, induces superior cross‐reactive CD8+ T‐cell responses and cross‐protection compared with mucosal vaccine administration. On the other hand, antibody responses induced by mucosal (IN) vaccination with WIV, in particular SIgA, can contribute to heterosubtypic cross‐protection in the absence of optimal flu‐specific CD8+ T‐cell immunity. Nonetheless, we conclude that parenteral vaccination is preferable for the induction of heterosubtypic cross‐protection against influenza using WIV. Currently used vaccines either lack conserved target antigens for CD8+ T cells (e.g., subunit vaccines) or lack intrinsic adjuvant components such as viral RNA (e.g., subunit and split virus vaccines) that could help to boost (cellular) immunity through TLR7/8 activation. WIV, however, contains both conserved target antigens for CD8+ T cells and TLR‐activating components and therefore holds promise as a candidate cross‐protective influenza vaccine for use in humans. The findings from this study may further guide the development and implementation of such a cross‐protective vaccine.
